# Surgically Diagnosed Diffuse Idiopathic Pulmonary Neuroendocrine Cell Hyperplasia in Asymptomatic Patients

**DOI:** 10.70352/scrj.cr.26-0123

**Published:** 2026-07-01

**Authors:** Chen Lu, Yosuke Matsuura, Ruxue Yang, Abigail Fickel, Guangyao Ning, Chenyu Sun

**Affiliations:** 1 Department of Thoracic Surgery, The First Affiliated Hospital of Anhui Medical University, Hefei, Anhui, China; 2 Department of Thoracic Surgical Oncology, Cancer Institute Hospital, Japanese Foundation for Cancer Research, Tokyo, Japan; 3 Department of Pathology, The First Affiliated Hospital of Anhui Medical University, Hefei, Anhui, China; 4 Mayo Clinic Alix School of Medicine, Mayo Clinic, Rochester, MN, USA; 5 Division of Public Health, Infectious Diseases, and Occupational Medicine, Mayo Clinic, Rochester, MN, USA; 6 Mayo Clinic School of Graduate Medical Education, Mayo Clinic College of Medicine and Science, Rochester, MN, USA; 7 School of Public Health, University of Minnesota-Twin Cities, Minneapolis, MN, USA

**Keywords:** diffuse idiopathic pulmonary neuroendocrine cell hyperplasia, pulmonary neuroendocrine tumors, mosaic attenuation, multiple pulmonary nodules

## Abstract

**INTRODUCTION:**

Diffuse idiopathic pulmonary neuroendocrine cell hyperplasia (DIPNECH) is a rare pulmonary precursor lesion that is characterized by the diffuse proliferation of neuroendocrine cells within the airway epithelium. Because obtaining a surgical specimen for histological confirmation is often difficult, management strategies for asymptomatic patients are controversial. It is generally considered an indolent condition that is often managed conservatively. However, diagnosis can be difficult in asymptomatic patients because radiological findings may resemble those of early-stage lung cancer.

**CASE PRESENTATION:**

We present 2 cases of asymptomatic patients with DIPNECH who were detected incidentally during radiological surveillance. High-resolution CT revealed multiple pulmonary nodules in both patients, accompanied by subtle findings indicative of small-airway involvement, such as mosaic attenuation and air trapping. Despite normal laboratory findings, normal pulmonary function tests, and the absence of respiratory symptoms, serial imaging revealed slow but definite enlargement of a dominant pulmonary nodule, making it difficult to exclude malignancy with confidence. The 1st patient was a 63-year-old nonsmoking woman who was observed for multiple pulmonary nodules over 6 years. During this period, a centrally located dominant nodule in the left lower lobe gradually enlarged, prompting a diagnostic left lower lobectomy. In the 2nd case, a 51-year-old nonsmoking woman demonstrated progressive enlargement of a dominant nodule over 2 years, prompting video-assisted thoracoscopic segmentectomy. Histopathological examination in both patients confirmed DIPNECH, allowing for the exclusion of invasive malignancy and a comprehensive pathological assessment of neuroendocrine cell proliferation.

**CONCLUSIONS:**

These cases illustrate a practical diagnostic challenge in patients with suspected DIPNECH who are asymptomatic. Although radiological surveillance is usually appropriate for indolent disease, the progressive enlargement of a dominant pulmonary nodule can limit the reliability of observation alone. Therefore, surgical resection can be justified as a diagnostic strategy within a multidisciplinary framework when malignancy cannot be confidently excluded, although careful patient selection and long-term surveillance remain essential.

## Abbreviations

CD56
: cluster of differentiation 56
CgA
: chromogranin A
DIPNECH
: diffuse idiopathic pulmonary neuroendocrine cell hyperplasia
HE
: hematoxylin–eosin
HRCT
: high-resolution CT
IHC
: immunohistochemical
INSM1
: insulinoma-associated protein 1
Ki-67
: Ki-67 antigen
MEN1
: multiple endocrine neoplasia type 1
PNETs
: pulmonary neuroendocrine tumors
Syn
: synaptophysin
TTF-1
: thyroid transcription factor-1
U-VATS
: uniportal video-assisted thoracoscopic surgery

## INTRODUCTION

DIPNECH is a rare but well-recognized pulmonary disorder characterized by diffuse proliferation of pulmonary neuroendocrine cells within the bronchial and bronchiolar epithelium.^1)^ Microscopically, DIPNECH is defined as a precursor lesion within the spectrum of PNETs and presents as multifocal neuroendocrine cell hyperplasia.^2)^ Lesions demonstrating invasion beyond the basement membrane are classified as carcinoid tumorlets when measuring less than 5 mm in diameter and as carcinoid tumors when measuring 5 mm or greater.^3)^

DIPNECH predominantly affects middle-aged, nonsmoking women, and diagnosis is frequently delayed due to nonspecific respiratory symptoms such as dry cough, dyspnea, and wheezing.^1)^ However, a subset of patients remains asymptomatic, and the disease may be detected incidentally on CT performed for unrelated reasons.^4)^ In such asymptomatic patients, diagnosis is particularly challenging because routine laboratory tests provide limited diagnostic value and clinical suspicion is often low.^5)^

HRCT plays a central role in the evaluation of suspected DIPNECH. Characteristic imaging findings include mosaic attenuation, air trapping, and multiple pulmonary nodules, which reflect underlying small-airway involvement.^6)^ Mosaic attenuation appears as patchy regions of differing attenuation on inspiratory and expiratory CT images and serves as an indirect indicator of bronchiolitis,^7)^ while multiple pulmonary nodules are typically bilateral, variable in number, and may demonstrate slow progression over time.^2)^ However, these radiological features are not disease-specific and may overlap with those of early-stage lung malignancy, particularly when a dominant pulmonary nodule demonstrates interval growth.^8,9)^

Hence, we present 2 cases of asymptomatic DIPNECH in which slow but definite enlargement of a dominant nodule prompted surgical resection. These cases underscore the importance of surgery in diagnosis when radiological findings alone cannot rule out malignancy.

## CASE PRESENTATION

The patients’ clinicopathological characteristics are summarized in **Table 1**.

**Table 1:** Clinico-imaging features

Items	Case 1	Case 2
Gender	Female	Female
Age (years)	63	51
Preoperative follow-up time (years)	6	2
Preoperative symptoms	Asymptomatic	Asymptomatic
Cigarette smoking status	Nonsmoker	Nonsmoker
Family history	None	None
Comorbidity	None	None
Surgical procedure	U-VATS, left lower lobectomy	U-VATS, left S4+5 segmentectomy
Laboratory examination	Normal	Normal
Pulmonary function	Normal	Normal
Location of the lesion	Left lower lobe	Left upper lobe
Number of nodules (dominant lesion excluded)	3	5
Mosaic attenuation on HRCT	Present (pronounced)	Present (mild)
Measurement of dominant lesion on HRCT (mm)	14.5 × 12.8 × 8.0	20.4 × 19.8 × 11.1
Gross morphological measurement of dominant lesion (mm)	16.0 × 12.0 × 7.0	20.0 × 17.1 × 6.0

HRCT, high-resolution CT; U-VATS, uniportal video-assisted thoracoscopic surgery

### Case 1

A 63-year-old nonsmoking woman was monitored for multiple pulmonary nodules that were detected incidentally. She remained asymptomatic throughout the 6-year observation period. Serial HRCT scans revealed a slow but definite enlargement of the largest nodule, which reached a maximum diameter of 14.5 mm. The lesion was centrally located in the left lower lobe, adjacent to the hilum. Mosaic attenuation and air trapping were observed on inspiratory and expiratory HRCT scans. Three additional small bilateral pulmonary nodules were also identified (**Fig. 1**).

**Fig. 1:**
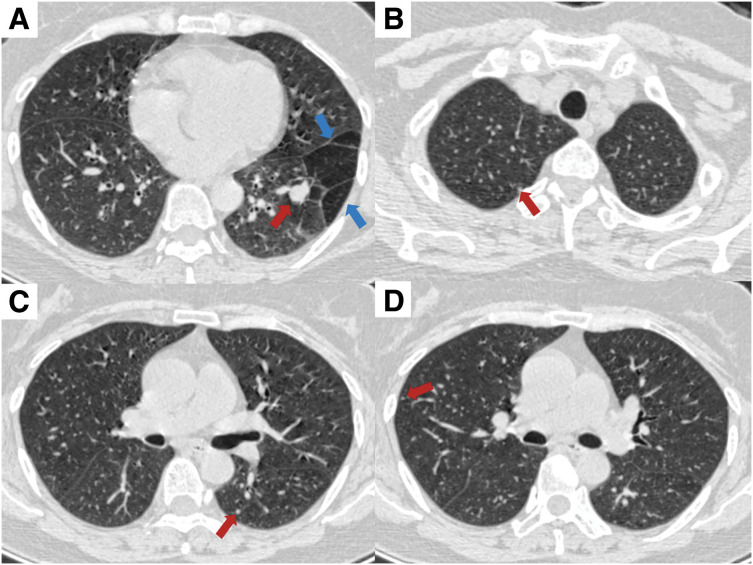
HRCT findings of case 1. HRCT of case 1 shows a dominant solid nodule in the left lower lobe (red arrow) with associated mosaic attenuation and air trapping (blue arrows) on inspiratory images (**A**). Additional small pulmonary nodules are observed in the right upper lobe, left lower lobe, and along the lateral lung fields (red arrows, **B**–**D**). HRCT, high-resolution CT

Laboratory examinations and pulmonary function tests were normal. Due to the interval growth of the dominant lesion, malignancy could not be confidently excluded, and surgical resection was recommended following a multidisciplinary discussion. Considering the anatomical location of the predominant lesion, CT-guided biopsy, endobronchial US-guided transbronchial needle aspiration (EBUS-TBNA) and transbronchial biopsy were considered technically challenging to yield sufficient tissue for a preoperative definitive diagnosis and wedge resection was not considered feasible (**Supplementary Figs. 1–3** and **Video 1**). Additionally, dense fissural fusion and adhesions identified limited the feasibility of sublobar resection intraoperatively. Therefore, left lower lobectomy via U-VATS was performed. Neuroendocrine proliferation was identified during the intraoperative frozen-section examination. However, a definitive diagnosis could not be made during the procedure.

Gross examination revealed a well-circumscribed lesion measuring 16.0 × 12.0 × 7.0 mm. Upon histopathological evaluation, low-magnification images demonstrated spatially separated, airway-centered neuroendocrine proliferations, rather than a single confluent tumor mass, with the largest focus measuring 2500 μm (**Fig. 2** and **Supplementary Figs. 4–7**). IHC staining was positive for TTF-1 (clone SP141), CD56, CgA, Syn, and INSM1. The Ki-67 index was approximately 1%, consistent with DIPNECH (**Fig. 3**). In addition, clinical manifestations, family history, and multi-organ endocrine tumors suggestive of MEN1 were absent in Case 1, thus excluding MEN1 from the differential diagnosis.

**Fig. 2:**
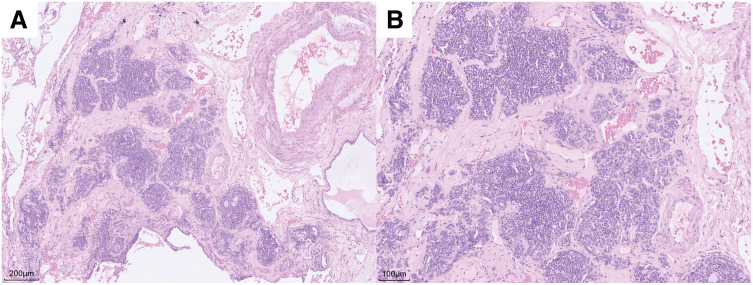
Histopathological findings of case 1. HE staining shows neuroendocrine cell proliferation forming tumorlets infiltrating beyond the bronchiolar basement membrane (**A**, ×100). The proliferating cells are relatively uniform, with salt-and-pepper chromatin and rare mitotic figures (**B**, ×200). HE, hematoxylin–eosin

**Fig. 3:**
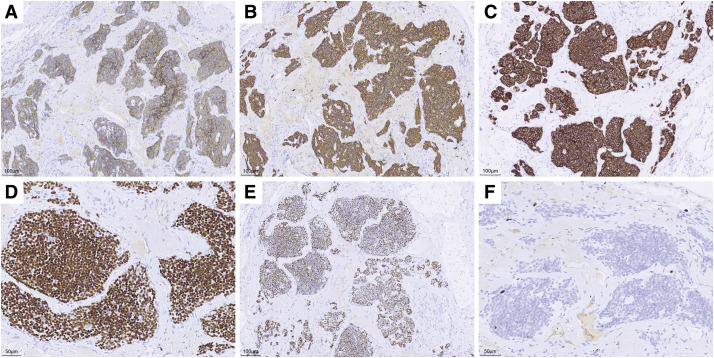
IHC findings of case 1. IHC staining demonstrates tumor cell positivity for CD56 (**A**, ×200), CgA (**B**, ×200), Syn (**C**, ×200), TTF-1 (**D**, ×400), and INSM1 (**E**, ×200). The Ki-67 proliferation index is approximately 1% (**F**, ×400). CD56, cluster of differentiation 56; CgA, chromogranin A; IHC, immunohistochemical; INSM1, insulinoma-associated protein 1; Ki-67, Ki-67 antigen; Syn, synaptophysin; TTF-1, thyroid transcription factor-1

The postoperative course was uneventful, and the patient was discharged on POD 13. At the 5-month follow-up, the remaining nodules were stable and no disease progression was observed.

### Case 2

A 51-year-old nonsmoking woman was found to have multiple pulmonary nodules during a routine health screening. She was asymptomatic, and her laboratory findings and pulmonary function tests were normal. HRCT revealed that a dominant pulmonary nodule had grown to 20.0 mm over a 2-year follow-up period, accompanied by mosaic attenuation and air trapping. Several small adjacent nodules and bronchiectasis-like changes were also noted (**Fig. 4** and **Supplementary Figs. 8, 9** and **Video 2**).

**Fig. 4:**
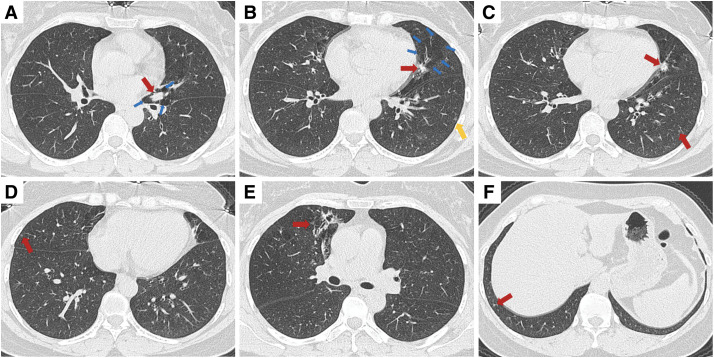
HRCT findings of case 2. HRCT of case 2 shows a dominant pulmonary nodule in left segments 4 and 5 (red arrow) with surrounding mosaic attenuation and air trapping (blue arrows) (**A**). Multiple adjacent small nodules and a bronchiectasis-like change are also noted (yerrow arrow, **B**; red arrows, **B–F**). HRCT, high-resolution CT

The dominant lesion was confined to segments S4 and S5. Therefore, S4+5 segmentectomy via U-VATS to diagnose was considered feasible while preserving uninvolved lung parenchyma. Since no definitive evidence of malignancy was found during the intraoperative frozen section examination and the resection margin was negative, no further resection was performed.

Gross examination revealed a well-defined lesion measuring 20.0 × 17.1 × 6.0 mm. Histopathological examination revealed multifocal neuroendocrine cell hyperplasia as in Case 1, with the largest focus measuring 3089 μm (**Fig. 5** and **Supplementary Figs. 10–12**). Tumor cells were positive for TTF-1 (clone SP141), CD56, CgA, Syn, and INSM1. The Ki-67 index was approximately 2%, which confirmed the diagnosis of DIPNECH (**Fig. 6**). As in case 1, MEN1 could be excluded from the differential diagnosis.

**Fig. 5:**
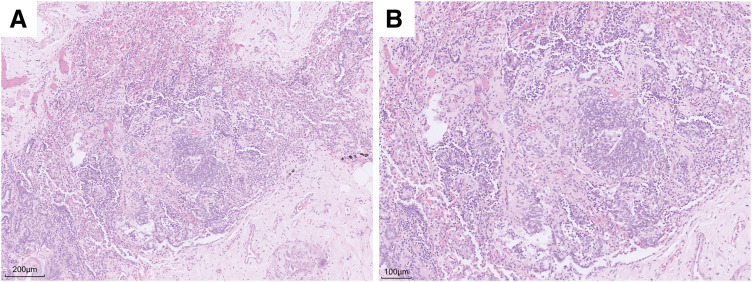
Histopathological findings of case 2. HE staining reveals hyperplastic neuroendocrine cells extending beyond the bronchiolar wall and forming nodular proliferations (**A**, ×100). The cells are small with faint cytoplasm and round to oval nuclei (**B**, ×200). HE, hematoxylin–eosin

**Fig. 6:**
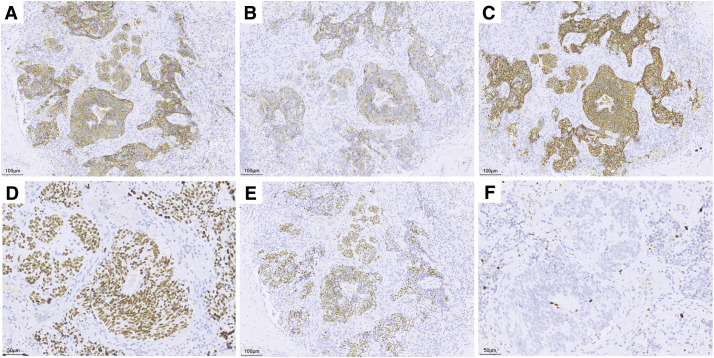
IHC findings of case 2. IHC staining demonstrated positivity for CD56 (**A**, ×200), CgA (**B**, ×200), Syn (**C**, ×200), TTF-1 (**D**, ×400), INSM1 (**E**, ×200), and Ki-67 (about 2%) (**F**, ×400). CD56, cluster of differentiation 56; CgA, chromogranin A; IHC, immunohistochemical; INSM1, insulinoma-associated protein 1; Ki-67, Ki-67 antigen; Syn, synaptophysin; TTF-1, thyroid transcription factor-1

The patient was discharged on POD 3 without complications. During the subsequent 10-month follow-up period, no recurrence or progression of residual nodules was observed.

## DISCUSSION

Although DIPNECH is a well-established pulmonary precursor lesion within the spectrum of PNETs,^1,3,10)^ its optimal management remains controversial, particularly in asymptomatic patients, and clear decision thresholds for surgical intervention in the presence of progressive dominant nodules have not been established. In clinical practice, such patients are often diagnosed incidentally during radiological surveillance and are generally managed conservatively because of the indolent nature of the disease.^11)^ However, when serial imaging demonstrates progressive enlargement of a dominant pulmonary nodule, clinicians face a diagnostic dilemma regarding whether continued observation is sufficient or whether surgical intervention is warranted for definitive diagnosis. The present cases highlight this under-recognized clinical scenario, in which surgical resection was performed primarily as a diagnostic strategy in asymptomatic patients with suspected DIPNECH.

HRCT plays a central role in raising suspicion for DIPNECH. Characteristic imaging findings include mosaic attenuation, air trapping, and multiple small pulmonary nodules, reflecting underlying small-airway involvement.^1,6,12)^ However, these findings are not disease-specific and may overlap with those of other small-airway disorders or early-stage lung malignancies.^8,9,13,14)^ A dominant pulmonary nodule that increases in size over time poses a particular diagnostic challenge, as radiological features alone may not reliably distinguish benign neuroendocrine proliferation from indolent malignancy.^1)^

In the cases detailed above, both patients were asymptomatic, and routine laboratory tests and pulmonary function assessments revealed no abnormalities. However, serial imaging revealed a slow but continuous enlargement of a dominant pulmonary nodule, accompanied by subtle yet characteristic findings of mosaic attenuation and air trapping indicative of small-airway involvement. These radiological changes raised concern for malignancy that could not be ruled out through noninvasive evaluation alone. Although DIPNECH itself is not an indication for surgical treatment,^1)^ the progressive nature of the dominant lesions prompted consideration of surgical intervention for a definitive diagnosis.^15)^

From a surgical perspective, these cases highlight a common practical dilemma in clinical decision-making. For asymptomatic patients with suspected DIPNECH, continued radiological surveillance is reasonable when lesions remain stable.^16)^ However, when a dominant nodule shows progressive enlargement, particularly when it is centrally located or associated with DIPNECH-like imaging features, the reliability of a diagnosis through observation alone becomes limited, and prolonged follow-up carries the risk of a delayed malignancy diagnosis. In such cases, because the minimally invasive biopsy techniques, such as transbronchial biopsy, EBUS-TBNA, or CT-guided biopsy were technically challenging or insufficient to provide a definitive pathological diagnosis, surgical resection was performed following multidisciplinary discussion. Surgery should not necessarily be regarded as a therapeutic intervention for DIPNECH itself, but rather as a diagnostic strategy when noninvasive evaluation cannot reliably exclude malignancy.

Although DIPNECH is generally considered an indolent disease, previous studies have shown that some patients develop clinically significant small-airway disease, most commonly constrictive bronchiolitis, which may result in chronic cough, dyspnea, and airflow restriction.^11)^ In addition, DIPNECH is regarded as a precursor lesion within the spectrum of PNETs, and progression to tumorlets or carcinoid tumors has been reported during long-term follow-up.^1)^ These observations suggest that the clinical course of DIPNECH may be heterogeneous. While surgical resection is not recommended as routine treatment for DIPNECH itself, it may be considered in selected cases when a dominant pulmonary nodule demonstrates progressive enlargement and malignancy cannot be confidently excluded by imaging or minimally invasive biopsy. In such situations, surgery can serve primarily as a diagnostic strategy allowing definitive histopathological evaluation.

Accordingly, surgical resection may be considered not as a therapeutic intervention for DIPNECH itself, but as a diagnostic strategy within a multidisciplinary framework.^17)^ As highlighted in these 2 cases, surgical resection allowed comprehensive histopathological evaluation, leading to a definitive diagnosis of DIPNECH and exclusion of invasive malignancy. Furthermore, resection allowed for accurate assessment of neuroendocrine cell proliferation, including evaluation of lesion size, distribution, and proliferative activity.

These cases also highlight the ongoing debate regarding the relationship between multifocal tumorlets and multifocal NETs in DIPNECH. Although tumorlets and carcinoid tumors are currently distinguished by a 5-mm size threshold according to the World Health Organization (WHO) criteria,^3)^ this classification may not fully reflect their underlying biological behavior. Increasing evidence suggests that DIPNECH, tumorlets, and carcinoid tumors may represent a spectrum of pulmonary neuroendocrine proliferation rather than entirely separate entities.^1,18)^ Whether the current size-based definition represents a true biological boundary or a practical histopathological criterion remains uncertain and warrants further investigation.^1)^ In the present cases, the largest neuroendocrine foci measured 2.5 and 3.1 mm, respectively, despite the substantially larger radiological appearance of the dominant lesions. According to the current WHO criteria, these findings support a classification of DIPNECH-associated tumorlets rather than carcinoid tumors. However, these cases also demonstrate the difficulty of distinguishing multifocal tumorlets from multifocal NETs based solely on size.

Although the long-term outcomes of surgically diagnosed, asymptomatic DIPNECH remain unclear, careful postoperative surveillance is warranted due to the potential for disease progression and the presence of residual pulmonary nodules.^1,18,19)^ These cases highlight the challenges of monitoring remaining nodules in asymptomatic patients with DIPNECH, as the disease is usually benign and typically managed conservatively. However, progressive enlargement of a dominant pulmonary nodule may limit the reliability of radiological surveillance alone. When imaging findings cannot confidently exclude malignancy and a minimally invasive biopsy is unlikely to provide a definitive diagnosis, surgical resection can be considered a rational diagnostic strategy rather than a therapeutic intervention. Previous studies have reported interval growth of pulmonary nodules in a proportion of patients, although the overall clinical course is usually indolent. Because standardized surveillance protocols have not yet been well established, periodic HRCT is generally recommended. In clinical practice, CT follow-up every 6–12 months during the initial postoperative surveillance period may represent a reasonable strategy for monitoring residual nodules.^1,20)^ Careful integration of subtle radiological findings, longitudinal imaging changes, and multidisciplinary discussion is essential to guide appropriate surgical decision-making in selected patients.

## CONCLUSIONS

Asymptomatic patients with radiological suspicion and uncertainty pose unique challenges in clinical diagnosis of DIPNECH. These cases highlight the valuable role of surgical resection in establishing a definitive diagnosis when progressive radiological changes raise concern for malignancy and nonsurgical biopsies are insufficient. Recognizing surgery as a diagnostic option within a multidisciplinary framework can help prevent both delayed diagnoses and unnecessary interventions. Continued postoperative surveillance is warranted given the potential for disease progression.

## SUPPLEMENTARY MATERIALS

Supplementary Fig. 1 Case 1. Axial chest CT image demonstrating the superior extent of the dominant lesion and its anatomical relationship to adjacent segmental bronchi.

Supplementary Fig. 2 Case 1. Axial chest CT image demonstrating the inferior extent of the dominant lesion and its anatomical relationship.

Supplementary Fig. 3 Case 1. Sagittal chest CT image demonstrating the inferior extent of the dominant lesion and its anatomical relationship.

Supplementary Fig. 4 Case 1. Low-magnification overview of the resected specimen.

Supplementary Fig. 5 Case 1. High-magnification overview of the resected specimen.

Supplementary Fig. 6 Case 1. Additional low-power image illustrating distortion of the normal alveolar architecture surrounding the neuroendocrine proliferations.

Supplementary Fig. 7 Case 1. Histopathological image showing fibrotic stroma and vascular remodeling between neuroendocrine proliferative foci.

Supplementary Fig. 8 Case 2. Axial chest CT image demonstrating the superior extent of the dominant lesion and its anatomical relationship to adjacent segmental bronchi.

Supplementary Fig. 9 Case 2. Sagittal chest CT image demonstrating the extent of the dominant lesion and its anatomical relationship to adjacent segmental bronchi.

Supplementary Fig. 10 Case 2. Low-magnification overview of the resected specimen.

Supplementary Fig. 11 Case 2. High-magnification overview of the resected specimen.

Supplementary Fig. 12 Case 2. Histopathological image showing fibrotic stroma and vascular remodeling between neuroendocrine proliferative foci.

Supplementary Video 1 Sequential axial chest CT images of Case 1 demonstrating the anatomical extent of the dominant lesion within the left lower lobe and its relationship to adjacent segmental bronchi.

Supplementary Video 2 Sequential axial chest CT images of Case 2 demonstrating the location of the dominant lesion within segments S4 and S5 and its relationship to surrounding anatomical structures.

## DECLARATIONS

### Availability of data and materials

Data will be made available on reasonable request.
