# Supplementary material for: Surgically Diagnosed Diffuse Idiopathic Pulmonary Neuroendocrine Cell Hyperplasia in Asymptomatic Patients
Source: Surg Case Rep. 2026 Jul 1;12(1):26-0123. doi: 10.70352/scrj.cr.26-0123 (PMC13329241; doi:10.70352/scrj.cr.26-0123)

Supplement figure 1

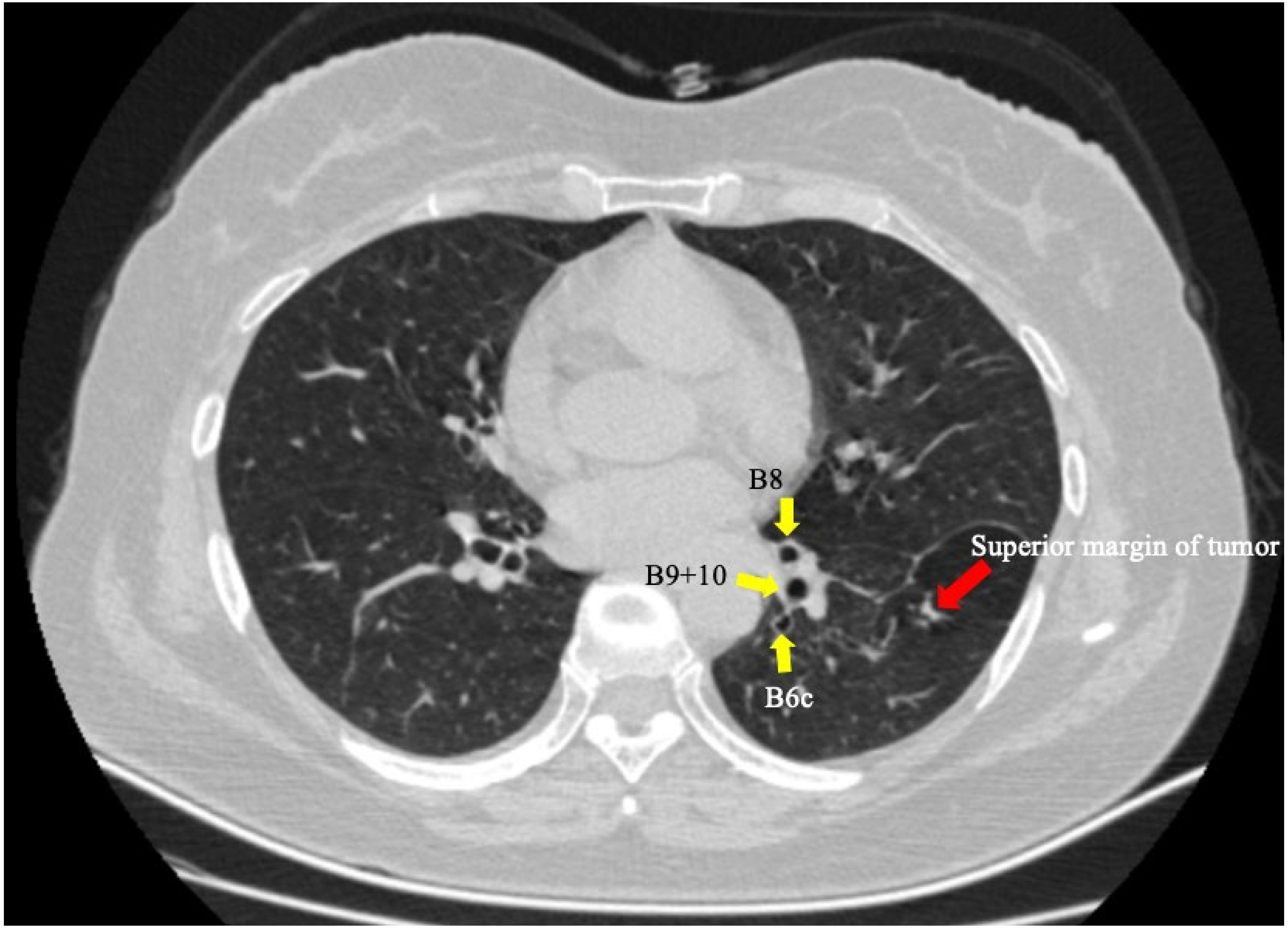

Supplement figure 2

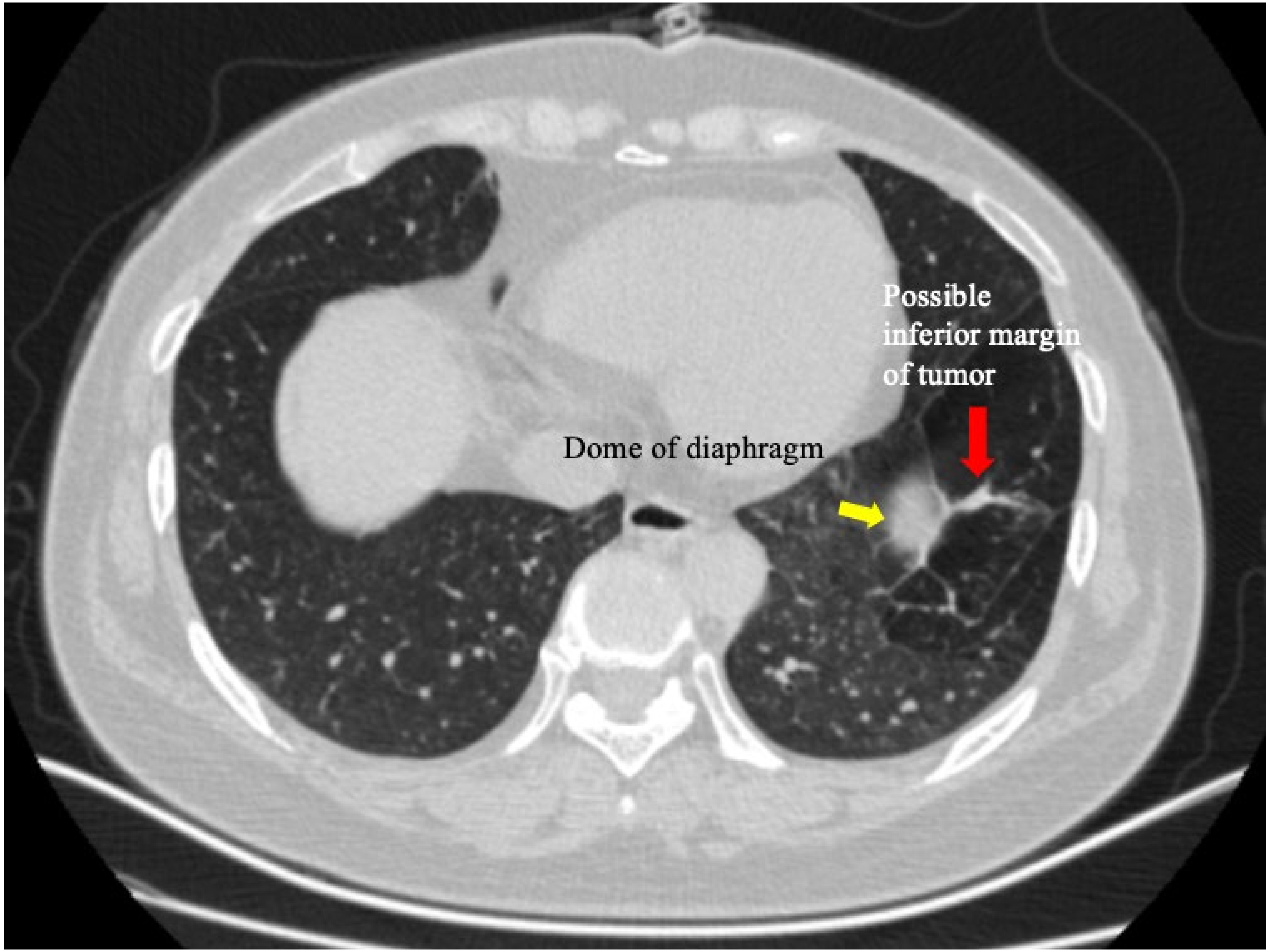

Supplement figure 3

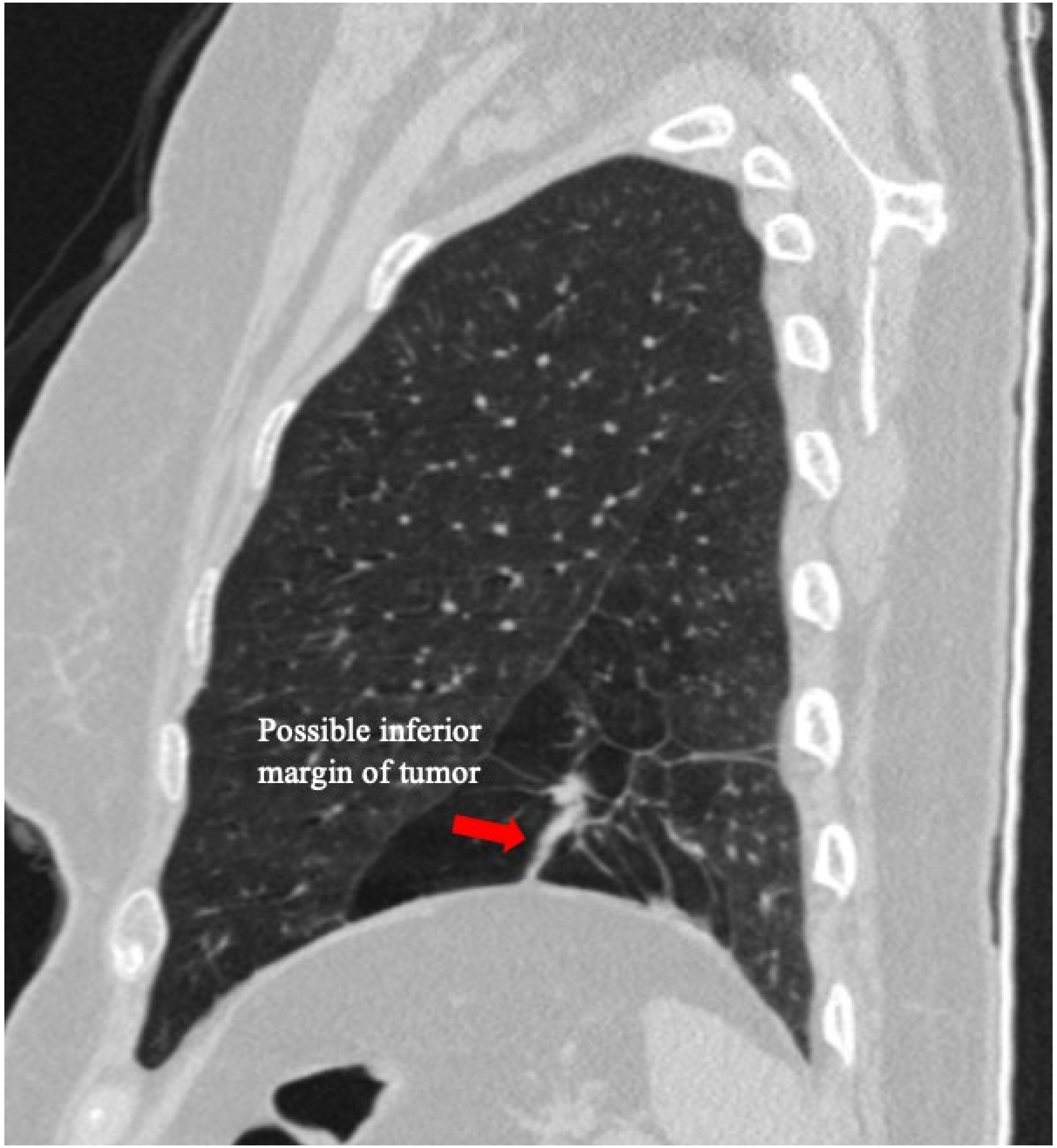

Supplement figure 4

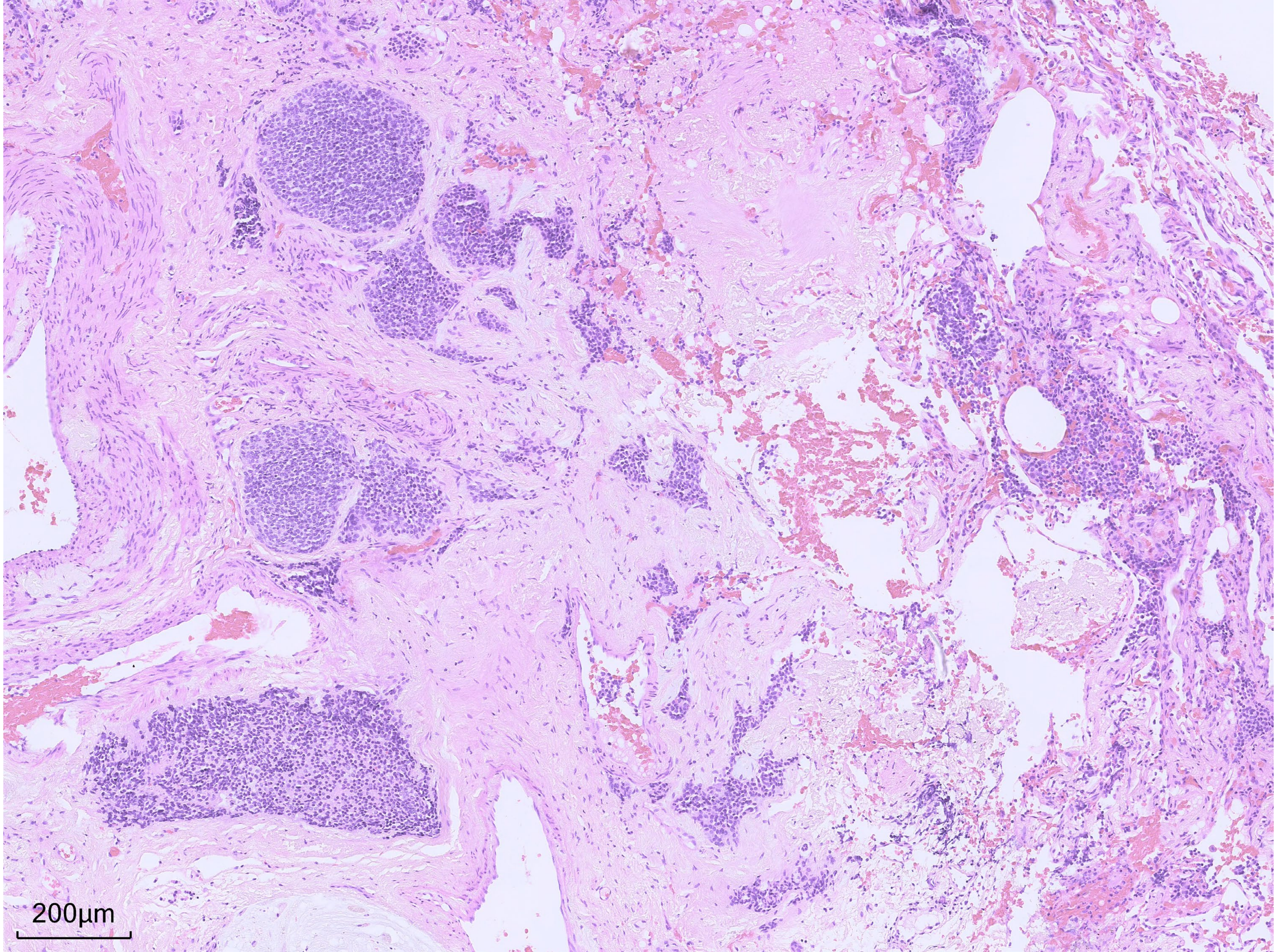

Supplement figure 5

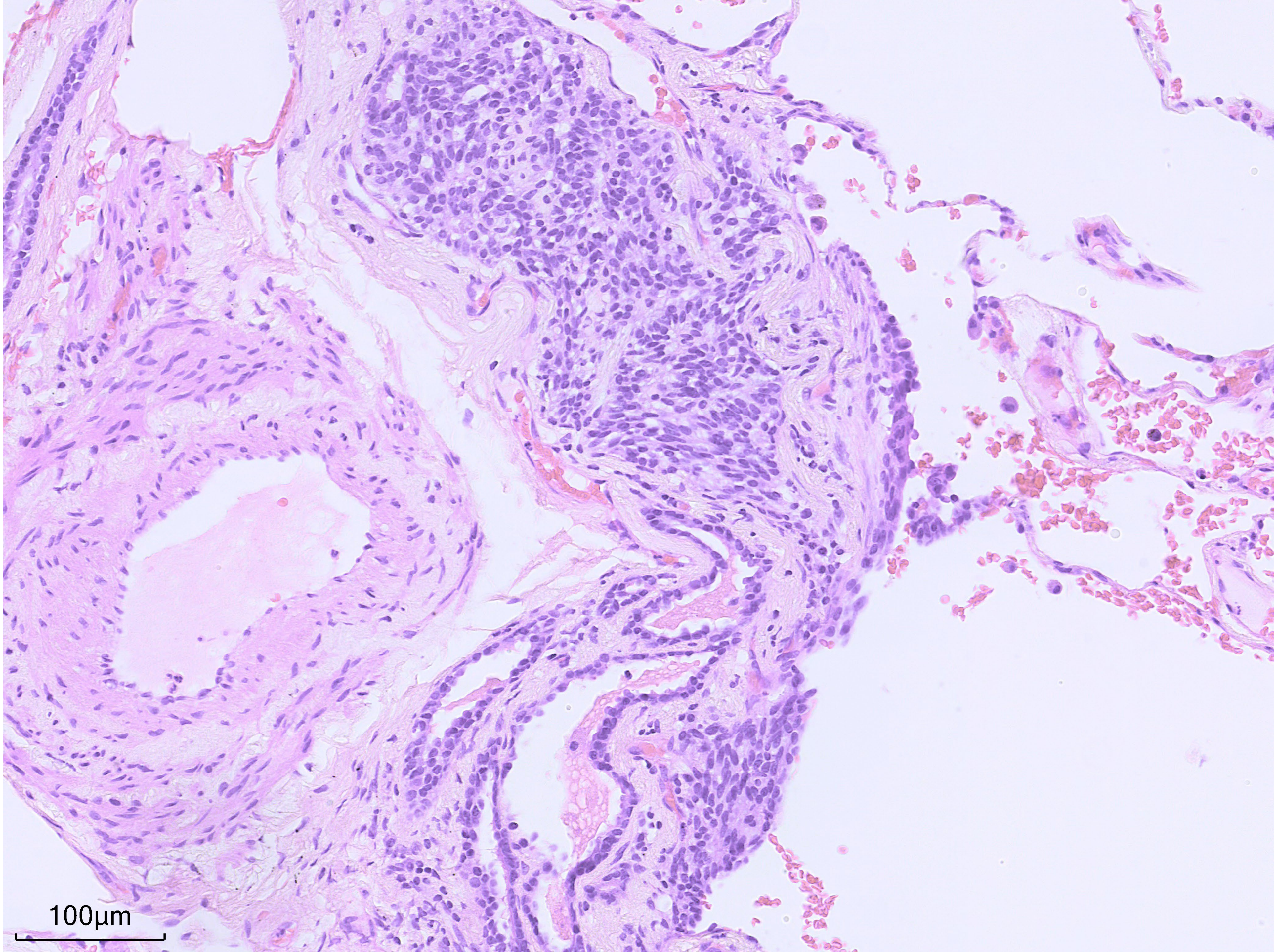

Supplement figure 6

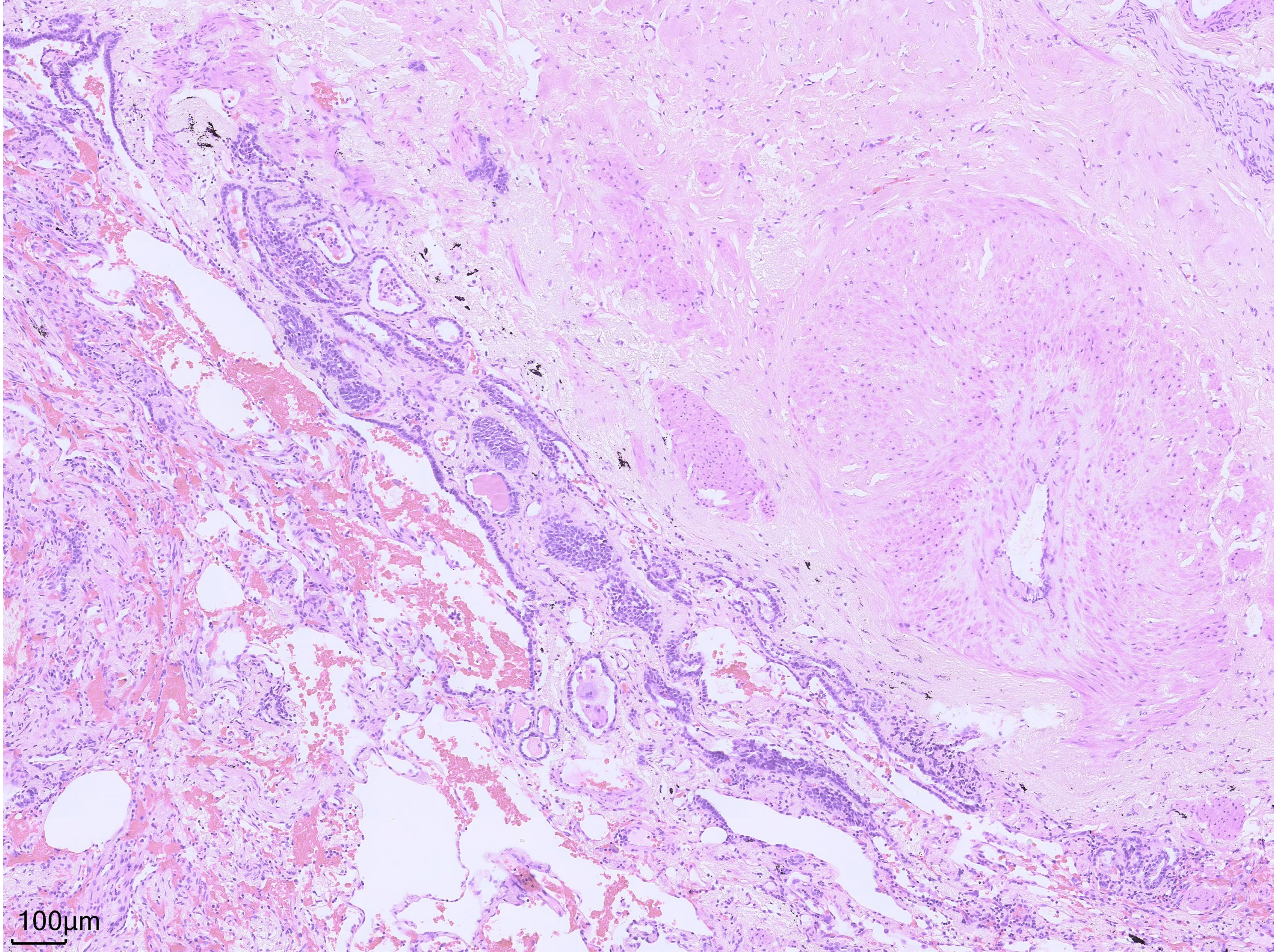

Supplement figure 7

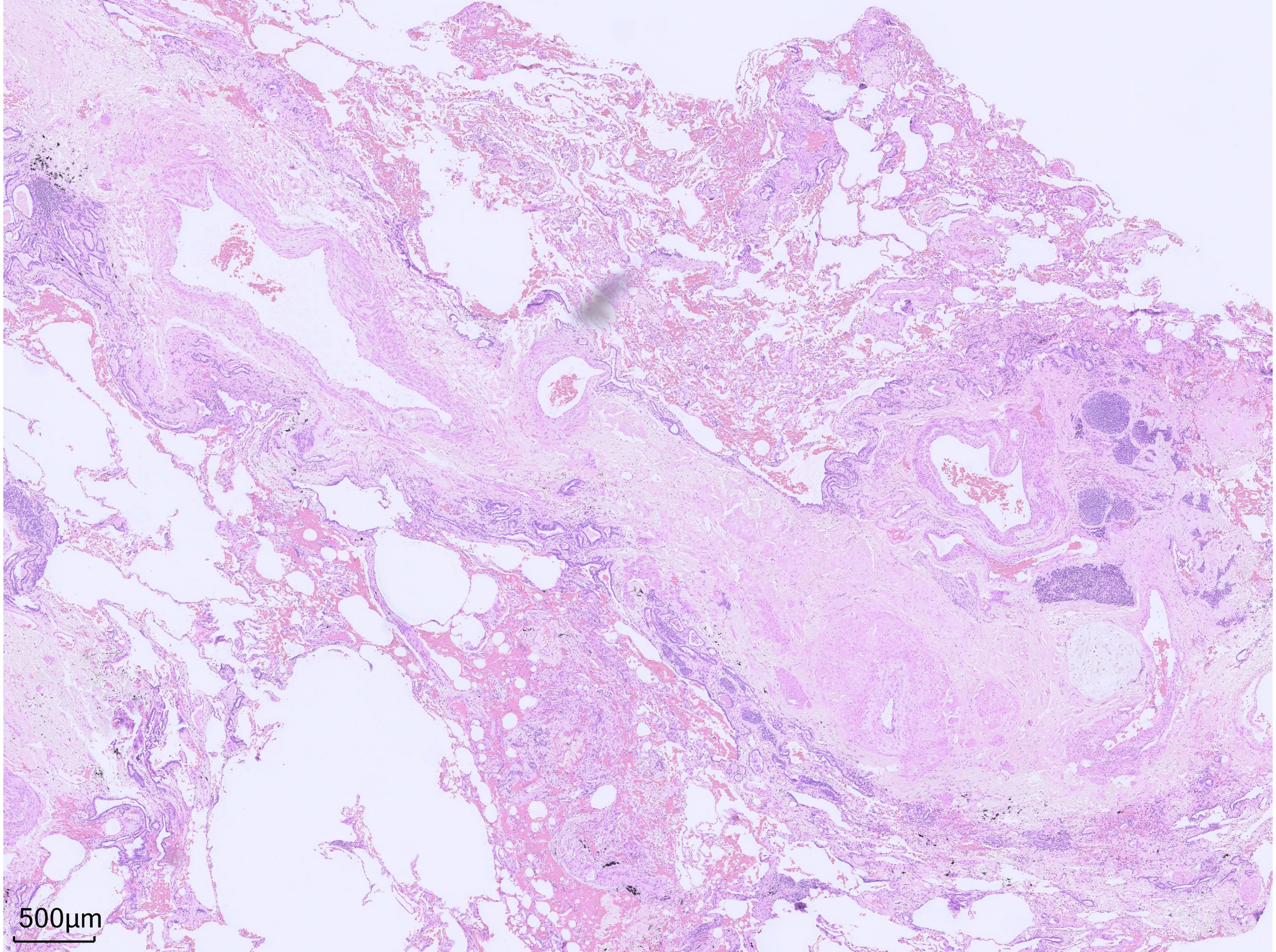

Supplement figure 8

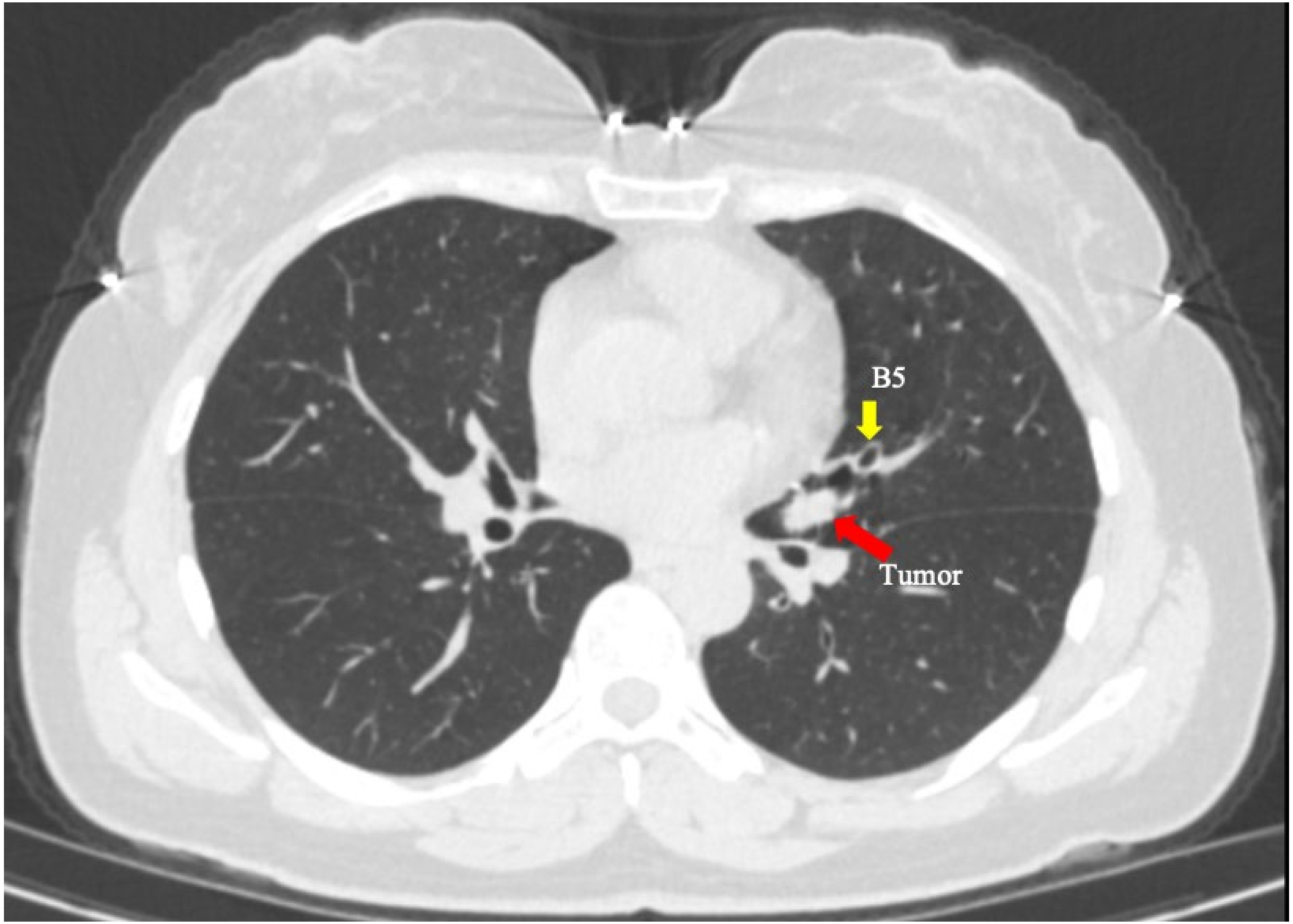

Supplement figure 9

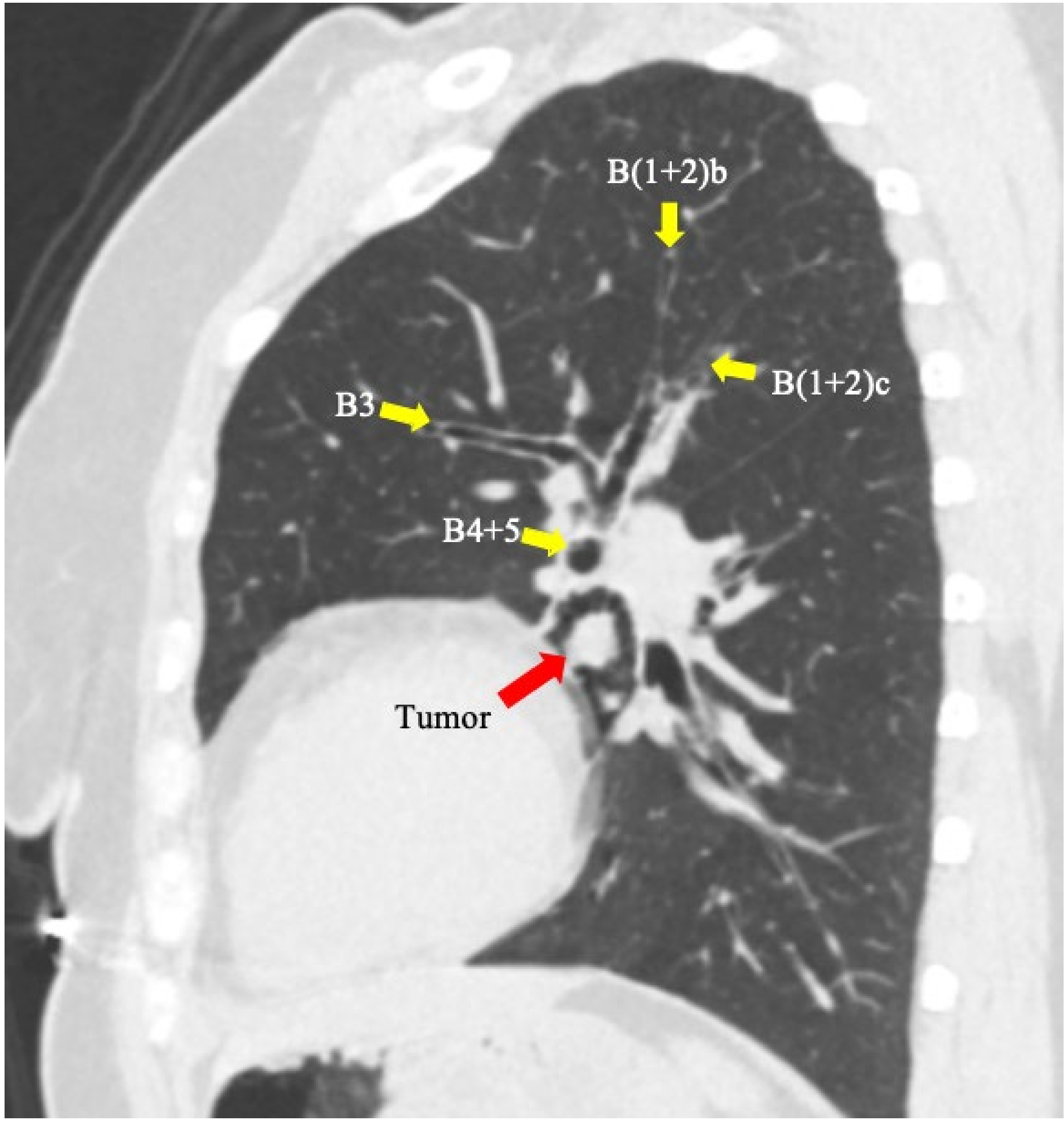

Supplement figure 10

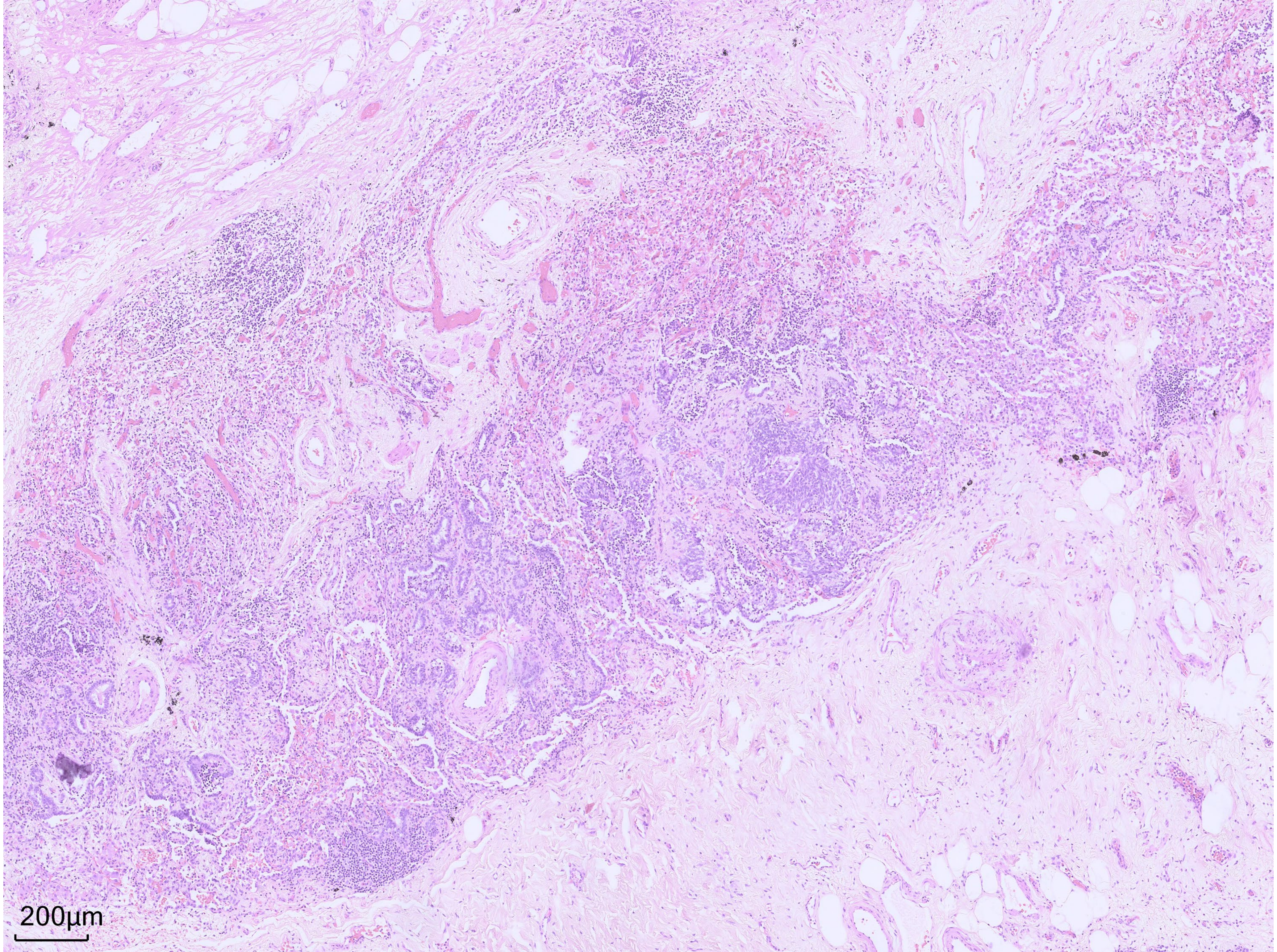

Supplement figure 11

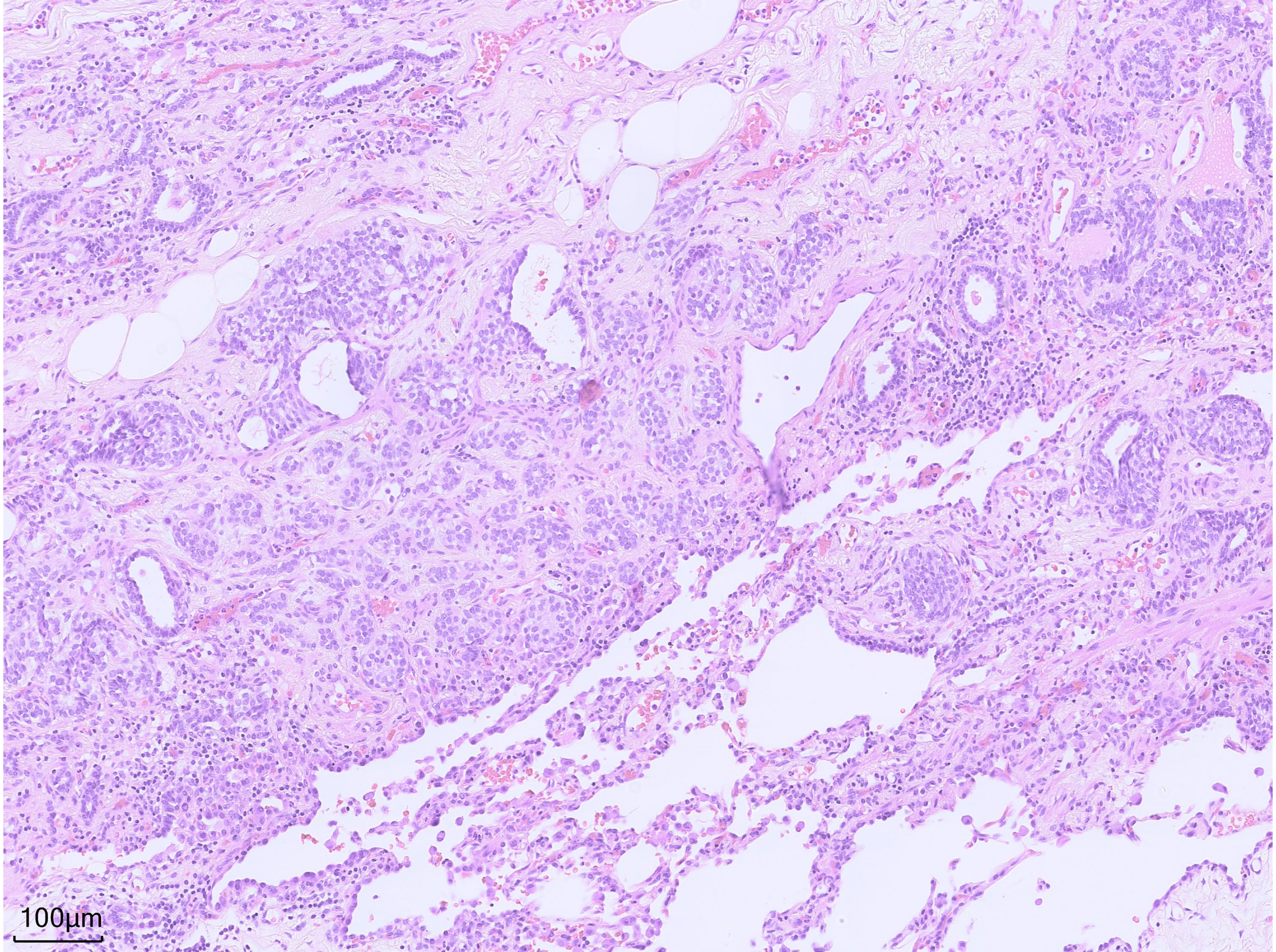

Supplement figure 12

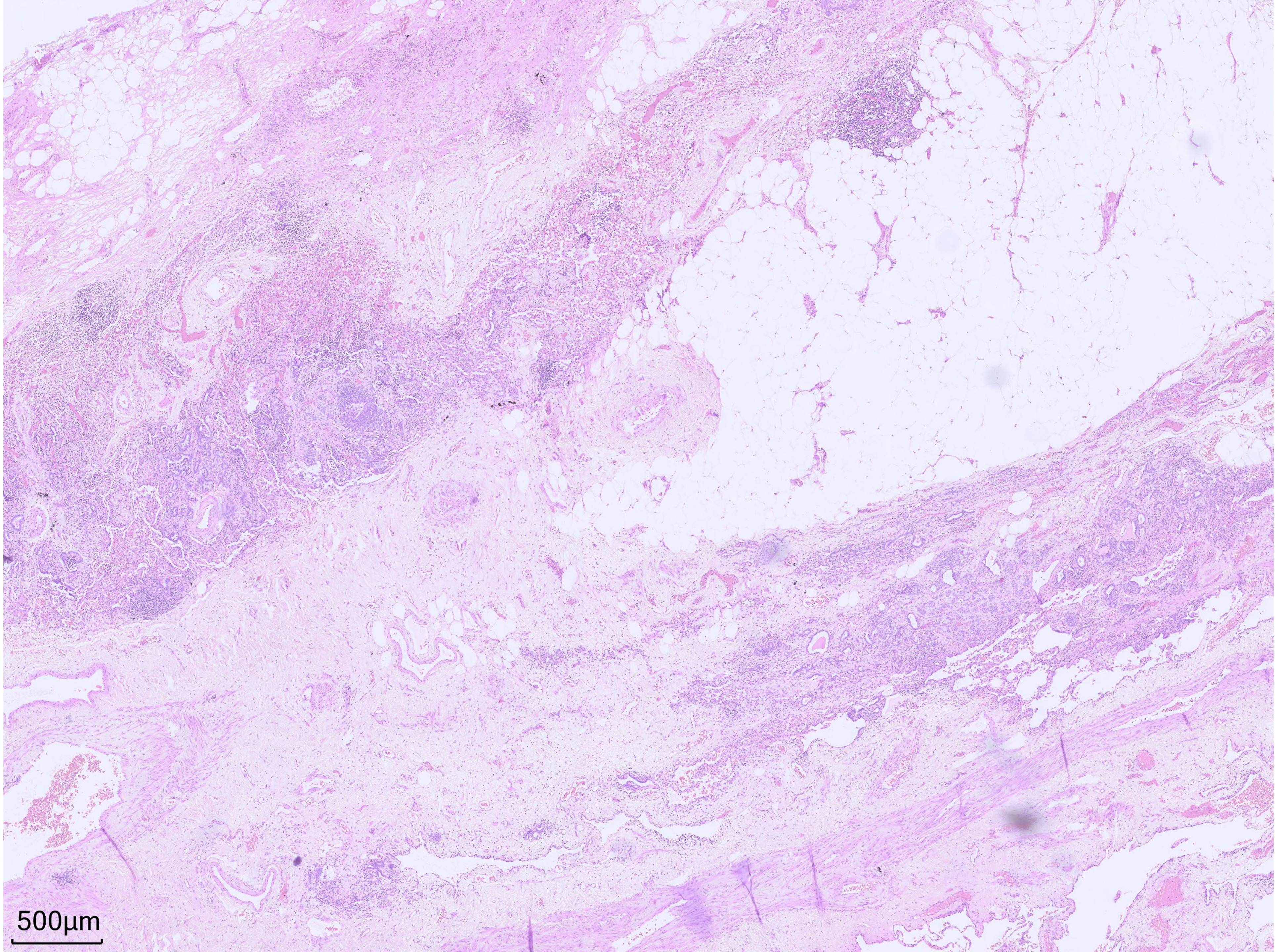

Supplement: Supplementary Fig. 1 — Case 1. Axial chest CT image demonstrating the superior extent of the dominant lesion and its anatomical relationship to adjacent segmental bronchi. [file scr-12-01-26-0123-s001.pdf]
